# Development of active edible coatings based on fish gelatin enriched with *Moringa oleifera* extract: Application in fish (*Mustelus mustelus*) fillet preservation

**DOI:** 10.1002/fsn3.2993

**Published:** 2022-07-22

**Authors:** Maram Mezhoudi, Ali Salem, Ola Abdelhedi, Nahed Fakhfakh, Mahmoud Mabrouk, Touhami Khorchani, Frederic Debeaufort, Mourad Jridi, Nacim Zouari

**Affiliations:** ^1^ University of Sfax, National Engineering School of Sfax Research Laboratory of Enzyme Engineering and Microbiology Sfax Tunisia; ^2^ Higher Institute of Applied Biology of Medenine University of Gabes Medenine Tunisia; ^3^ Arid Regions Institute of Medenine Central Laboratory Medenine Tunisia; ^4^ Arid Regions Institute of Medenine Research Laboratory of Livestock and Wild Life Medenine Tunisia; ^5^ Univ. Bourgogne Franche‐Comté/AgrosupDijon, UMR PAM A02.102 Physical‐Chemistry of Food and Wine Lab Dijon France; ^6^ Institut Universitaire de Technologie de Dijon, BioEngineering Department Dijon Cedex France; ^7^ University of Jendouba Higher Institute of Biotechnology of Beja Beja Tunisia

**Keywords:** antioxidant activity, bioactive gelatin coating, *Moringa oleifera*, quality, quinic acid, smooth‐hound fillet

## Abstract

An edible coating was developed using gelatin extracted from the skin of gray triggerfish (*Balistes capriscus*) and applied to the fillet of the smooth‐hound shark (*Mustelus mustelus*). *Moringa oleifera* leaf extract was added to gelatin coating solution to improve its preservative properties. The phenolic profiles and antioxidant and antibacterial activities of *M. oleifera* extracts were determined. Phenolic acids constituted the largest group representing more than 77% of the total compounds identified in the ethanol/water (MOE/W) extract, among which the quinic acid was found to be the major one (31.48 mg/g extract). The MOE/W extract presented the highest DPPH• scavenging activity (IC_50_ = 0.53 ± 0.02 mg/ml) and reducing (Fe^3+^) power (EC_0.5_ = 0.57 ± 0.02 mg/ml), as well as interesting inhibition zones (20–35 mm) for the most tested strains. Coating by 3% of gelatin solution significantly reduced most deterioration indices during chilled storage, such as malondialdehyde (MDA), total volatile basic nitrogen (TVB‐N), weight loss, pH, and mesophilic, psychrophilic, lactic, and H_2_S‐producing bacterial counts. Interestingly, coating with gelatin solution containing MOE/W extract at 20 μg/ml was more effective than gelatin applied alone. Compared with the uncoated sample, gelatin‐MOE/W coating reduced the weight loss and MDA content by 26% and 70% after 6 days of storage, respectively. Texture analysis showed that the strength of uncoated fillet increased by 46%, while the strength of fillet coated with gelatin‐MOE/W only increased by 12% after 6 days of storage. Fish fillet coated with gelatin‐MOE/W had the highest sensory scores in terms of odor, color, and overall acceptability throughout the study period.

## Graphical abstract

Quinic acid was found to be the major compound in the ethanol/water Moringa extract gelatin coating enriched with Moringa extract contributed to delay its deterioration

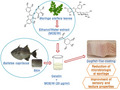

## 1 INTRODUCTION

Currently there is growing interest in using ecological food packaging based from natural polymers as an alternative to conventional nonbiodegradable polymers. Moreover, the development of “active packaging” by incorporating bioactive compounds with antimicrobial and antioxidant activities into the polymer matrices continues to develop progressively until today. This active packaging can be interesting to preserve perishable food products and thus improve their shelf‐life and sensory quality (Araghi et al., 2015; Li et al., 2019; Abdelhedi et al., 2019; Eghbalian et al., 2021; Shahbazi et al., 2021).

Fish gelatin, produced by partial hydrolysis of collagen, is an interesting alternative to mammalian‐derived gelatin for the production of edible packaging films. In fact, it can be obtained economically from fish by‐products, without consumer concern, as in the case of porcine and bovine gelatin. Fish gelatin, which was tasteless and colorless, has excellent film‐forming, biocompatibility, and mechanical properties especially in the presence of certain agents, such as cross‐linking adhesion promoters, other proteins and polysaccharides, among others, that improve its functional properties (Alfaro et al., 2014). Besides, gelatin formed an excellent matrix for hosting bioactive compounds, such as plant extract rich in phenolic compounds or essential oils that can be used to develop antioxidant and antimicrobial packaging films (Hanani et al., 2019; Naeeji et al., 2020; Shahbazi et al., 2021; Staroszczyk et al., 2020). In this context, edible gelatin‐based films or coatings have been used to preserve foods, such as trout fillets (Eghbalian et al., 2021), cheese (Salem et al., 2021), shrimp (Mirzapour‐Kouhdasht & Moosavi‐Nasab, 2020), bread (Oliveira et al., 2020), and beef meat (Jridi et al., 2018), in order to improve their quality during storage.

*Moringa oleifera* Lam. (Moringaceae) that is called the tree of life was originally from India and is widely cultivated in many parts of the world, such as Tunisia, since it adapts to arid and heat‐resistant regions. It has been considered as one of the most useful perennial trees due to its high content of nutrients and bioactive compounds without any reported undesirable side effects. The leaves have been used to treat malnutrition and have also been used as fortificant in many food products (Oyeyinka & Oyeyinka, 2018). Besides, several pharmacological properties of *M. oleifera* have been reported, such as hepatoprotective, neuroprotective, antidiabetic, anti‐inflammatory, anticancer, and antiviral activities (Gopalakrishnan et al., 2016; Razzaq et al., 2020). However, few studies have been reported regarding the use of this plant into biopolymer matrices.

The use of edible coatings based on gelatin and natural bioactive compounds would be an interesting strategy for improving the quality of food products. Thus, *M. oleifera* was chosen as a potential plant of bioactive substances. The phenolics profiles and antioxidant and antibacterial activities of different solvent extracts from *M. oleifera* leaves were determined. Gelatin was extracted from the skin of gray triggerfish (*Balistes capriscus*) and then was used to develop gelatin‐based coatings enriched with *M. oleifera* extracts. The effect of active gelatin coating on the physicochemical and microbiological quality of smooth‐hound shark (*Mustelus mustelus*) fillets during refrigerated storage was studied.

## 2 MATERIAL AND METHODS

### 2.1 Plant material

*Moringa oleifera* Lam. (Moringaceae) leaves were collected from the oasis of Chenini‐Gabes (southeastern Tunisia, characterized by an arid climate) on April 2020. The leaves were air‐dried in the shadow, until constancy of the mass (20 days), then ground into fine powder, and stored at ambient temperature in a dry place and in the dark until use.

### 2.2 Phytochemical analysis and antioxidant activities

The *M. oleifera* leaves were extracted using three solvents: (i) ethanol 100%, (ii) ethanol/water (50/50, v/v), and (iii) distilled water. Leaves powder (5 g) was macerated in 100 ml of each solvent in a closed Erlenmeyer flask and stirred at 250 rpm for 12 h. Then, the macerate was filtered through Whatman No. 1 filter paper. The same procedure was repeated twice with the obtained residue, and then the extract was lyophilized and kept in the dark at +4°C until further analysis. Finally, three extracts were obtained:

- ethanol extract (MOE);
- ethanol/water (50/50, v/v) extract (MOE/W);
- water extract (MOW).

After that, the total phenolics and flavonoids in *M. oleifera* extracts were measured as described previously (Dewanto et al., 2002). The total phenolics content was expressed as mg gallic acid equivalent (GAE)/g extract. The flavonoids content was expressed as mg catechin equivalent (CE)/g extract. *Moringa oleifera* extracts were also analyzed using liquid chromatography–electrospray ionization–tandem mass spectrometry (LC‐ESI‐MS) as described previously by Jdir et al. (2017). An LC‐MS‐2020 quadrupole mass spectrometer (Shimadzu, Kyoto, Japan) equipped with an electrospray ionization source and operated in negative ionization mode was used. The identification of phenolics was done by comparing the retention times and the mass spectra with those of authentic standards of highest purity (≥99.0%), which were from Sigma Chemical Co. (St. Louis, MO, USA).

The reducing (Fe^3+^) power and DPPH• radical‐scavenging activity of *M. oleifera* extracts were measured as described previously (Yıldırım et al., 2001; Zouari et al., 2011). Results of DPPH• radical‐scavenging activity were presented by IC_50_ values, defined as the extract concentration needed to scavenge 50% of DPPH•. In the reducing power assay, the presence of antioxidants in the sample would result in the reduction of Fe^3+^ to Fe^2+^, which can be monitored by measuring the formation of Perl's Prussian blue (Fe_4_[Fe(CN)_6_]_3_) at 700 nm. The extract concentration providing an absorbance of 0.5 at 700 nm (EC_0.5_) was presented. Lower IC_50_ and EC_0.5_ values reflected better antioxidant activities. All tests were carried out in triplicate and the results were averaged.

### 2.3 Antimicrobial activity

#### 2.3.1 Bacterial strains

Antibacterial activities of *M. oleifera* extracts were tested against eight bacteria strains: 4 g‐negative (*Escherichia coli* ATCC 25922, *Pseudomonas aeruginosa* ATCC 27853, *Salmonella enterica* ATCC 43972, and *Enterobacter aerogenes*) and 4 g‐positive (*Bacillus cereus* ATCC 11778, *Staphylococcus aureus* ATCC 25923, *Micrococcus luteus* ATCC 4698, and *Listeria monocytogenes*). Microorganisms were obtained from the culture collection of the Biotechnology Center of Sfax, Tunisia.

#### 2.3.2 Agar well diffusion method

Antibacterial activity of *M. oleifera* extracts was determined using agar well diffusion method of Vanden Berghe and Vlietinck (1991). Culture suspension (200 μl) of the tested microorganisms (10^6^ colony forming units [CFU]/ml of bacteria cells) were spread on the Mueller Hinton broth (MHB) and three wells of 0.5 cm deep were made by using a sterile tip. Then, 60 μl of each extract (1 mg/ml) was added to respective wells. Gentamycin was used as positive reference and the negative control was done with sterile water. Prior to incubation, all plates were stored in the dark at 4°C for 2 h to allow diffusion of the extract to the medium without bacterial growth. At the end of incubation period (24 h at 37°C), the antimicrobial activity was determined by measuring the zone of inhibition around the holes in diameter (mm) after incubation. All tests were carried out for three sample replications and the results were averaged.

### 2.4 Gelatin extraction and coating preparation

The by‐product of gray triggerfish (*B. capriscus*) was obtained after processing the fish from the Sfax market (Sfax, Tunisia). Gelatin was extracted from fish skin as described previously by Jellouli et al. (2011). The skin was washed with tap water and cut into small pieces (1 × 1 cm) and then soaked in 0.05 M NaOH solution at a ratio of 1:5 (m/v). The mixture was stirred for 2 h and the alkaline solution was changed every 30 min. Then, the alkaline‐treated skin was washed with distilled water until a neutral pH was obtained, and then subjected to acid treatment, at a ratio of 1:5 (m/v), using few drops of acetic acid to reach a pH of 3.0 over 18 h with gentle stirring. After that, the pH was neutralized using few drops of 6 M NaOH solution and the mixture was incubated at 50°C with continuous stirring for 24 h. Finally, the mixture was centrifuged at 6000*g* for 20 min to remove insoluble matter and gelatin‐containing supernatant was freeze‐dried using a freeze‐dryer Christ Alpha 1‐2 (Bioblock Scientific, Illkirch, France) and stored at 4°C until use. The gelatin coating solution was prepared by mixing 3 g of dried gelatin in 100 ml of distilled water at 40°C for 30 min. In order to prepare the active gelatin solution, the MOE/W extract was dissolved in the gelatin solution at a final concentration of 20 μg/ml.

### 2.5 Preparation of fish fillet samples

Fresh smooth‐hound shark (*Mu. mustelus*) fillets were purchased from a local fish market (Sfax, Tunisia). Fish fillets were cut into 2 × 2 × 2 cm cubes and divided into three groups according to the following treatments: (i) fillet: uncoated fish fillet; (ii) fillet + gelatin: fish fillet coated with a control gelatin solution; and (iii) fillet + gelatin‐MOE/W: fish fillet coated with gelatin solution enriched with *M. oleifera* ethanol/water extract at 20 μg/ml. Fish fillets were coated with gelatin solution during 30 s at 50°C. After treatment, all samples were weighed and stored at 4°C without packaging to simulate retail setting environment. Microbial, physical, and chemical analyses of the different experiments were realized during 6 days of refrigerated storage.

### 2.6 Characterization of coated fish fillets

#### 2.6.1 pH measurement

Two grams of fish fillet sample were homogenized in 20 ml of distilled water for 2 min. Then, the pH was measured using a pH meter (Hanna Instruments, Póvoa de Varzim, Portugal). The pH of fish samples from each treatment was measured after 1, 2, 4, and 6 days of storage.

#### 2.6.2 Weight loss

The weight loss of fish fillet was calculated using the Equation (1).

(1) $$\text{Weight loss}\;\left(\%\right)=\frac{W_{0}-W_{i}}{W_{0}}\times 100$$

where *W*_0_ is the initial weight of fish sample and *W*_*i*_ is the weight of the same sample after 1, 2, 4, and 6 days of storage.

#### 2.6.3 Color measurement

Color was measured using a colorimeter (Konica Minolta, Osaka, Japan) with D65 illuminant. The instrument was standardized using a standard white plate. Color was measured for fish samples from each treatment after 1 and 6 days of storage. The CIELAB color space was used to obtain the color coordinates of L* (lightness scale varying from 0 [black] to 100 [white]), a* (scale varying from −*a* [green] and +*a* [red]), and b* (scale varying from −*b* [blue] and +*b* [yellow]). From these parameters, the total difference (∆*E*) and the saturation (C*) in color of the fish fillet samples were determined using Equations (2) and (3).

(2) $$\text{∆}E=\sqrt{{\left(L^{*}-L_{c}^{*}\right)}^{2}+{\left(a^{*}-a_{c}^{*}\right)}^{2}+{\left(b^{*}-b_{c}^{*}\right)}^{2}}$$

(3) $$C^{*}=\sqrt{{a^{*}}^{2}+{b^{*}}^{2}}$$

where L*, a*, and b* are the color parameters of the fish samples; $L_{c}^{*}$, $a_{c}^{*}$, and $b_{c}^{*}$ are the color parameters of the uncoated fish fillet samples on the first day of storage.

#### 2.6.4 Texture profile analysis (TPA)

The TPA parameters (strength, cohesiveness, springiness, and chewiness) were measured according to the method described previously by Jridi et al. (2015) using a texture analyzer (Lloyd Instruments, Ltd., West Sussex, UK). The samples were cut into small cubes of 2 × 2 cm on both sides. TPA was determined according to the program: pretest speed: 0.5 mm/s; test speed: 5 mm/s; and trigger force: 0.05 N. The fish sample was subjected to two cycle's compression up to 30% of its original height using a 12‐mm diameter cylindrical probe. The measurement was performed in triplicate.

#### 2.6.5 Total volatile basic nitrogen (TVB‐N)

The TVB‐N was measured after perchloric acid distillation from homogenized fish fillet samples (Abelti, 2013). The distillate was recovered in an Erlenmeyer flask containing aqueous solution of 20 g/L boric acid and some drops of methyl red as an indicator. Then, the boric acid solution was titrated with 0.1 M sulfuric acid solution. The TVB‐N (mg N/100 g sample) was measured based on the volume of sulfuric acid used for titration according to the following Equation (4).

(4) $$\mathrm{TVB}-N\left(\frac{\mathrm{mg}}{100}g\right)=\frac{V\;x\;N\;x\;14\;}{m}\times 100$$

where *V* is the volume of sulfuric acid consumed for titration, *N* is the normality of the sulfuric acid, and *m* is the sample mass.

#### 2.6.6 Lipid peroxidation

The thiobarbituric acid reactive substances (TBARS) of fish samples were measured as described previously by Witte et al. (1970). Briefly, 5 g of fish sample was homogenized in 20 ml of 5% trichloroacetic acid solution using a Polytron PT 2100 homogenizer (Kinematica AG, Luzern, Switzerland) for 5 min. The homogenate was centrifuged at 10,000 *g* for 10 min at 4°C. The supernatant (4 ml) was reacted with 0.8 ml 0.6 M chlorhydric acid and 3.2 ml Tris‐thiobarbituric acid (TBA) solution (26 mM Tris, 120 mM TBA) and then incubated in a water bath at 85°C for 10 min. The absorbance of each mixture was measured at 532 nm. TBARS values were calculated from a standard curve of malondialdehyde (MDA) and expressed as mg MDA/kg fish sample.

#### 2.6.7 Microbiological analysis

Bacteriological counts were measured by mixing 1 g of fish sample in 9 ml of 0.9% NaCl solution, then appropriate decimal dilutions were prepared. The mesophilic and psychrotrophic counts were measured using plate count agar medium. The inoculated plates were incubated at 37°C for 2 days for the mesophilic bacteria and at 4°C for 7 days for the psychrotrophic bacteria. Iron and de Man, Rogosa, and Sharpe agar were used to enumerate H_2_S‐producing bacteria (incubation at 37°C for 48 h) and lactic acid bacteria (LAB) (incubation at 30°C for 72 h). All bacterial counts were converted to logarithms of colony‐forming units per gram of fish fillet (log_10_ CFU/g) (Nowzari et al., 2013).

#### 2.6.8 Sensory analysis

Sensory evaluation of fresh fish samples was performed by 30 panelists who give a score for color (10 = no discoloration; 1 = extreme discoloration), odor (10 = extremely like; 1 = extremely unacceptable/off‐odors), and overall acceptability (10 = extremely like; 1 = extremely unacceptable). For each analysis day, fillet piece for each treatment was placed in the individual booths, which had a random three‐digit blind code and presented in the unsystematic order (Naeeji et al., 2020).

### 2.7 Statistical analysis

One‐way analysis of variance (ANOVA) was done using the statistical package for the social sciences (SPSS) software for Windows™ (version 17, SPSS Inc., Chicago, IL, USA). Duncan's multiple range test was used to compare the measured responses for different fish samples. Differences between means at the 95% (*p* ≤ .05) confidence level were considered statistically significant.

## 3 RESULTS AND DISCUSSION

### 3.1 Characterization of *M. oleifera* leaves

#### 3.1.1 Bioactive compounds profile

The bioactive compounds of *M. oleifera* leaves were extracted using three solvents: ethanol, ethanol/water (50/50, v/v), and water. Total phenolics and flavonoids contents were presented in Table 1. The ethanol/water extract showed the highest content of total phenolics (90.13 mg GAE/g extract) and flavonoids (16.77 mg QE/g extract) as compared to the ethanol and water extracts. Prabakaran et al. (2018) reported comparable levels of total phenolics (90–112 mg GAE/g extract) and flavonoids (55–69 mg QE/g extract) in alcoholic and aqueous extracts of *M. oleifera* leaves.

**TABLE 1:** *Moringa oleifera* leaves extract: LC‐ESI‐MS analysis, polyphenolic and flavonoid contents, and antioxidant activities. The DPPH• radical scavenging of each identified compound is presented by the concentration that inhibits 50% of the DPPH• radical (IC_50_) according to the literature

No^a^	Compounds^b^	Molecular formula	Molecular mass	[M‐H]^−^ m/z	Retention time (min)	Extract content (μg/g extract)			IC_50_ (μg/ml)
						MOE	MOE/W	MOW	
1	Quinic acid	C_7_H_12_O_6_	192	191	2	1511	31,484	490	>191^c^
2	Gallic acid	C_7_H_6_O_5_	170	169	4.5			36,260	0.7^d^
3	Protocatechuic acid	C_7_H_6_O_4_	154	153	6.5	711	268.6	588	0.9^e^
4	Caffeic acid	C_9_H_8_O_4_	180	179	14.8	124.4	62.9	39.2	2.4^f^
5	*p*‐Coumaric acid	C_9_H_8_O_3_	164	163	21.2	240	182.8	107.8	105^g^
6	*Trans*‐ferulic acid	C_10_H_10_O_4_	194	193	23.3	44.4	34.3		3.4^d^
7	Quercetin‐3‐*O*‐galactoside	C_21_H_20_O_12_	464	463	24.8	5422	5485	29.4	5.2^h^
8	Quercetin‐3‐*O*‐rhamonoside	C_21_H_20_O_11_	448	447	26.8	3822	3657	19.6	12.5^i^
9	Naringin	C_27_H_32_O_14_	580	579	26.4	26.7	34.3		>116^j^
10	*Trans*‐cinnamic acid	C_9_H_8_O_2_	148	147	32.1	222.2			96.7^k^
11	Quercetin	C_15_H_10_O_7_	302	301	32.2	44.4	5.7	9.8	10.57^i^
12	Naringenin	C_15_H_12_O_5_	272	271	34.2	8.9	5.7	29.4	272^i^
13	Luteolin	C_15_H_10_O_6_	286	285	35.2	26.7	11.4	9.8	5.1^f^
14	Cirsiliol	C_17_H_14_O_7_	330	329	35.9	106.7	91.4	127.4	2.3^f^
Total of identified compounds (mg/g extract)						12.31	41.32	37.71	
Total phenolics (mg GAE/g extract)						23.20 ± 1.0	90.13 ± 2.1	49.41 ± 1.3	
Flavonoids (mg QE/g extract)						4.49 ± 0.2	16.77 ± 1.0	12.04 ± 0.3	
Reducing (Fe^3+^) power (EC_0.5_, mg/ml)						1.08 ± 0.04	0.57 ± 0.02	0.70 ± 0.1	
DPPH• scavenging activity (IC_50_, mg/ml)						0.94 ± 0.1	0.53 ± 0.02	0.65 ± 0.06	

*Note*: MOE, MOE/W, and MOW represent ethanol, ethanol/water (50/50, v/v), and water extracts from *M. oleifera* leaves, respectively. Total phenolics content as gallic acid equivalent (GAE); flavonoids content as quercetin equivalent (QE). Reducing power is expressed by EC_0.5_ value that corresponds to the extract concentration providing an absorbance of 0.5 at 700 nm. DPPH• scavenging activity is expressed by IC_50_ value, defined as the extract concentration needed to scavenge 50% of DPPH•.

^a^ The numbering refers to elution order of compounds from an Aquasil C18 column.

^b^ Identification was confirmed using 32 authentic commercial standards.

^c^ Izuta et al. (2009).

^d^ Mishra et al. (2012).

^e^ Kakkar and Bais (2014).

^f^ Yokozawa et al. (1998).

^g^ Ayranci and Erkan (2013).

^h^ Zhao et al. (2013).

^i^ Khanduja and Bhardwaj (2003).

^j^ Luis and Johnson (2005).

^k^ Chethan et al. (2008).

The LC‐ESI‐MS analysis of *M. oleifera* extracts was also assessed and profiles of phenolic compounds are shown in Table 1. Fourteen compounds distributed into seven phenolic acids (compounds 1–6 and 10) and seven flavonoids were identified by comparing the obtained mass spectra with those of 32 authentic standards of phenolic compounds. However, if the analyzed extracts contained different compounds from the used standards, they cannot be identified.

Table 1 shows that quinic acid, gallic acid, quercetin‐3‐*O*‐galactoside, and quercetin‐3‐*O*‐rhamnoside were the major compounds measured in different extracts. Moreover, the content of some compounds varied considerably from one extract to another, which suggest the important effect of the solvent nature on their extraction. The phenolic acids constituted the largest group accounting more than 99% of the total identified compounds in aqueous extract, among which the gallic acid (36.26 mg/g extract) was the major one. The ethanol/water solution was the better solvent for quinic acid extraction (31.48 mg/g extract). However, the flavonoids were the most extracted molecules in ethanolic extract representing 77% of the total identified compounds. Recently, we have studied the profiles of phenolic compounds from the same leaf sample, but dried at 50°C (Mezhoudi et al., 2022). Similar profiles were obtained, but with lower contents for quinic acid (13.5 mg/g extract) and gallic acid (6.8 mg/g extract). In fact, the increase in temperature during oven drying (50°C) showed a decrease in quinic and gallic acids by 57% and 81%, respectively, which could be attributed to the heat sensitivity of these compounds. Similar results were described by Bettaieb Rebey et al. (2020) who reported that oven drying (65°C) caused the degradation of free phenolic acids in anise seeds (*Pimpinella anisum*), while shade drying (18°C) resulted in relatively high concentrations in phenolic compounds.

Anwar et al. (2007) reported many flavonoids in *M. oleifera* leaves with predominance of the kaempferol and quercetin in their 3‐*O*‐glycoside forms. However, the occurrence and content of these metabolites depend on the edaphoclimatic conditions (Wink, 2003). Besides, Nobossé et al. (2018) reported that the contents of bioactive compounds and antioxidant activity of *M. oleifera* leaves were influenced by the plant age and the extraction solvent used. It was reported that mixture of alcohol/water or acetone/water are the best extraction solvents with respect to the extraction yields of polar phenolic acids (Stalikas, 2007).

Quinic acid is a cyclic polyalcohol representing an important biochemical intermediate of the shikimate pathway, involved in the biosynthesis of aromatic compounds in plants (Herrmann & Weaver, 1999). Consumption of quinic acid as a dietary supplement was reported to increase the synthesis of nicotinamide and tryptophan in the gastrointestinal tract, which inhibits nuclear factor kappa B (NF‐κB) and improves DNA repair (Pero et al., 2009). In addition, quinic acid exhibits powerful antioxidant, hepatoprotective, anti‐inflammatory properties, as well as other interesting medicinal properties (Pero et al., 2009; Xiang et al., 2001). In the same context, it has been suggested that quinic acid could be used as a potent drug candidate against prostate cancer (Inbathamizh & Padmini, 2013).

#### 3.1.2 Antioxidant potential

The abundance of phenolic compounds in *M. oleifera* leaves could confer an interesting antioxidant potential, since these compounds have the ability to stabilize the free radical generation by their reactivity as electron‐ or hydrogen‐donating molecules. Thus, the extracts were subjected to their antioxidant activities, which were evaluated by reducing (Fe^3+^) power and DPPH• radical‐scavenging assays (Table 1). The antioxidant activities of *M. oleifera* extracts in the different assays increased with increasing extract concentration in a dose‐dependent manner (data not shown). Table 1 shows that ethanol/water extract presented the highest reducing (Fe^3+^) power and DPPH• scavenging activity, since it presented the lowest EC_0.5_ and IC_50_ values. A survey of the literature shows that the majority of the identified compounds had potent antioxidant potential with IC_50_ values in the DPPH• radical‐scavenging assay less than 15 μg/ml (Table 1). Jaiswal et al. (2013) concluded that the high content of phenolic compounds in the *M. oleifera* leaves can provide protection against oxidative damage in normal and diabetic subjects. Besides, Kushwaha et al. (2014) reported that 60 postmenopausal women fed over a 3‐month period with *M. oleifera* leaf powder reduced the malondialdehyde level and improved the serum‐produced antioxidant enzymes activities in response to lipid peroxidation, which favors the plant's antioxidant potential. Thus, consumption of *M. oleifera* or food products supplemented with the leaves of this plant is likely to provide antioxidant potential and consequently health benefits as reported previously (Singh et al., 2020).

#### 3.1.3 Antibacterial properties

The antibacterial activity of *M. oleifera* extracts against eight species of microorganisms was evaluated by the inhibition zones determination (Table 2). The different extracts presented varying degrees of antimicrobial activity against the tested strains. The highest inhibition zones for the most strains were obtained for the ethanol/water and water extracts, which suggest that these extracts may be useful in inhibition of many microorganisms. Prabakaran et al. (2018) reported the antimicrobial activity of five solvent extracts from different parts of *M. oleifera* against *P. aeruginosa* and *Erwinia carotovora*. They reported that among all the solvents, the methanol and ethyl acetate extracts showed interesting inhibition zones against both strains, while the aqueous and acetone extracts showed limited inhibition.

**TABLE 2:** Antibacterial activities of *Moringa oleifera* extracts

Strains	Inhibition zone diameters (mm)			
	MOE	MOE/W	MOW	Gentamycin*
Gram −				
*Escherichia coli*	17 ± 0.5^a^	22 ± 1.0^b^	22 ± 0.5^b^	21.0 ± 1.0^b^
*Pseudomonas aeruginosa*	13 ± 1.0^a^	33 ± 1.5^b^	34 ± 0.5^b^	14.0 ± 2.0^a^
*Salmonella enterica*	16 ± 1.0^a^	15 ± 1.0^a^	23 ± 0.5^b^	14.0 ± 2.0^a^
*Enterobacter aerogenes*	23 ± 0.5^a^	14 ± 1.0^b^	20 ± 0.5^c^	16.0 ± 2.0^b^
Gram +				
*Micrococcus luteus*	14 ± 1.5^a^	28 ± 1.3^b^	26 ± 0.5^b^	18.0 ± 1.0^c^
*Staphylococcus aureus*	12 ± 0.5^a^	19 ± 1.0^b^	20 ± 0.5^b^	37.0 ± 1.0^c^
*Bacillus cereus*	17 ± 1.0^a^	17 ± 1.0^a^	16 ± 0.5^a^	22.0 ± 2.0^b^
*Listeria monocytogenes*	16 ± 1.0^a^	35 ± 2.5^b^	35 ± 0.5^b^	18.0 ± 1.0^a^

*Note*: MOE, MOE/W, and MOW represent ethanol, ethanol/water (50/50, v/v), and water extracts from *M. oleifera* leaves, respectively. Different lower case letters (^a,b,c^) in the same line indicate significant differences between the different samples (*p* ≤ .05).

* Gentamycin (10 μg/well) was used as positive control for bacteria.

The presence of many phenolic compounds may explain the observed antimicrobial activity. In fact, Fu et al. (2016) reported that various phenolic compounds (gallic acid, caffeic acid, ferulic acid, quercetin, and luteolin), which were identified in the studied extracts, have interesting antimicrobial activity. The antibacterial activity of phenolic compounds should include many mechanisms, such as disruption of cell membrane structure and permeability and inhibition of enzymes necessary for nucleic acid synthesis leading to cell death (Gill & Holley, 2006; Gradišar et al., 2007). Besides, one can notice that synergistic and antagonistic effects between several phenolic compounds in the extract should also be taken into consideration.

Thus, phenolic compounds identified in *M. oleifera* leaves could be used as a potential natural antibacterial and antioxidant agent to control food poisoning diseases as well as oxidation. In the following, the ethanol/water extract was chosen as a potential extract to be added in the gelatin solution used to coat the smooth‐hound shark fillets.

### 3.2 Quality evaluation of coated fish fillets

#### 3.2.1 Physicochemical characteristics

**FIGURE 1:**
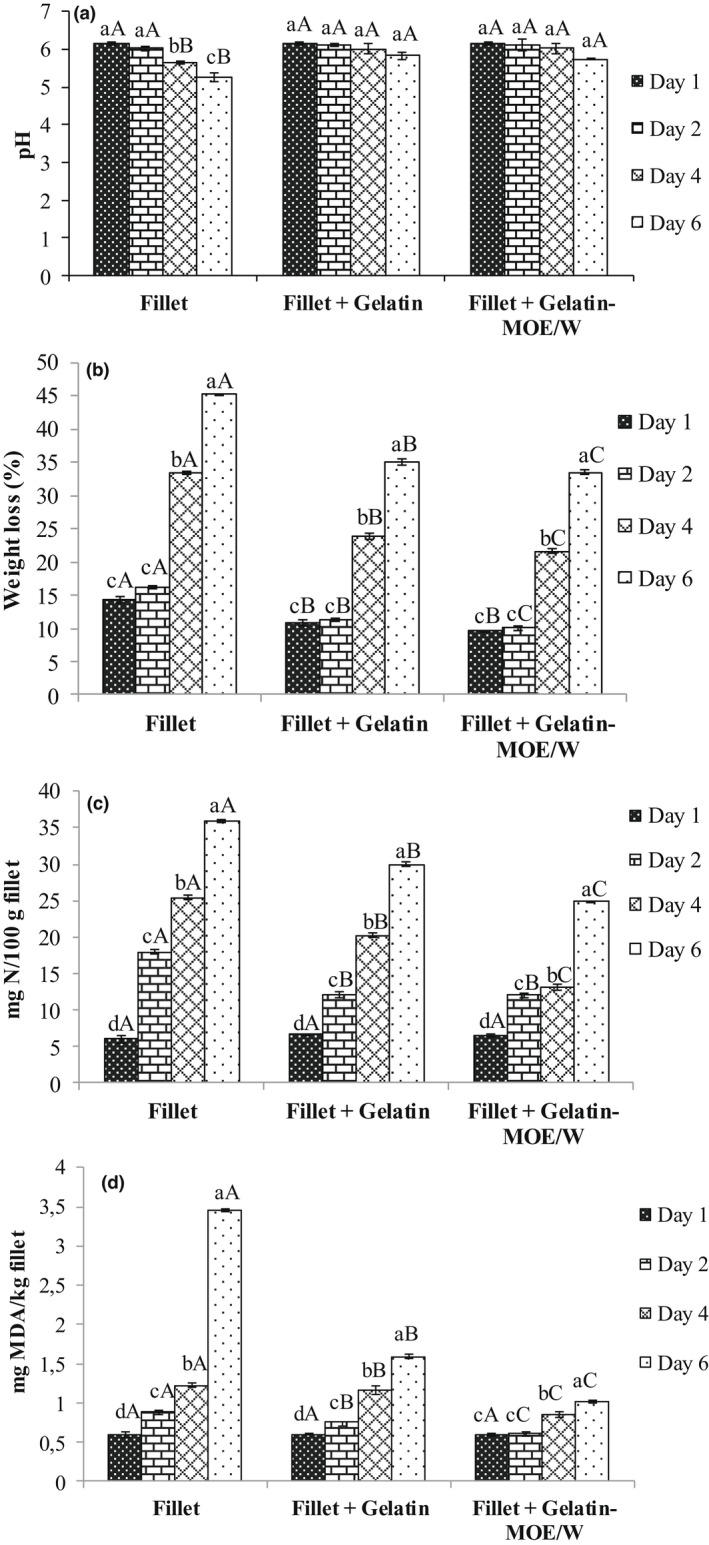
Changes in (a) pH, (b) weight loss, (c) total volatile basic nitrogen, and (d) lipid peroxidation of coated fish samples during storage. Fillet: uncoated fish fillet; fillet + gelatin: fish fillet coated with the control gelatin solution; fillet + gelatin‐MOE/W: fish fillet coated with gelatin solution enriched with *Moringa oleifera* ethanol/water extract at 20 μg/ml. Different lower case letters (^a,b,c^) indicate significant differences for the same sample within different days of storage (*p* ≤ .05). Different capital letters (^A,B,C^) indicate significant differences between samples at the same storage day (*p* ≤ .05)

Figure 1 shows the effect of fish fillets coating with gelatin on the evolution of pH, weight loss, TVB‐N, and lipid peroxidation during 6 days of storage. Initially, the pH of all fish samples was 6.16, then it significantly decreased (*p* ≤ .05) at the end of the storage period for all samples. Interestingly, this decrease was significantly lower (*p* ≤ .05) for gelatin‐coated samples, which may suggest its beneficial effect on maintaining the quality of the fish fillets during storage. The pH decrease could be explained by the production of lactic acid (Lorenzo et al., 2014). However, Abdelhedi et al. (2019) reported an increase in pH values at the beginning of the refrigerated storage period of fish fillets, which could be due to the formation of alkaline compounds by microbiological activity or endogenous enzymes.

Figure 1 also shows that syneresis, evaluated by the weight loss measurement, significantly increased (*p* ≤ .05) during storage for the different fish samples. Syneresis was more marked for the uncoated fish fillet, which could be induced by proteins denaturation of fish tissues, as well as by the absence of an outer packaging. Syneresis was significantly reduced (*p* ≤ .05) by coating the fish fillet with gelatin. Interestingly, the enrichment of gelatin with *M. oleifera* extract further reduced syneresis. In fact, the coating with gelatin and gelatin‐MOE/W decreased the weight loss by 22% and 26% after 6 days of storage, respectively, as compared to the uncoated sample. Similarly, Feng et al. (2016) showed the importance of gelatin coating in controlling the moisture loss from fish fillets during cold storage. The observed decrease in syneresis in gelatin‐coated samples could be explained by the water‐holding capacity of gelatin. In the case of coating with gelatin‐MOE/W, phenolic compounds could improve water retention and prevent moisture loss outside the fish fillet. Indeed, the hydroxyl groups present in some phenolics could bind water. It was reported that natural plant phenolic compounds were used for structural modification of gelatin. Araghi et al. (2015) reported that caffeic acid was an effective phenolic compound that improves the barrier and physicochemical properties of gelatin packaging (Araghi et al., 2015).

The TVB‐N developed in fish products during storage are widely used as chemical indicator of their spoilage, with a critical limit of 25–35 mg N/100 g fish fillet (Šimat et al., 2009). The initial TVB‐N was determined to be 6.05–6.16 mg/100 g, which was close to the value (8.12 mg N/100 g) reported for fresh trout fillets (Eghbalian et al., 2021). Figure 1 shows that TVB‐N content gradually increased for the different fish samples during 6 days of storage. The uncoated sample exceeded the critical freshness limit on the fourth day, while the sample coated with gelatin maintained its quality on the fourth day. Interestingly, the sample coated with gelatin‐enriched *M. oleifera* extract did not exceed the critical TVB‐N limit throughout the storage period. The increase in TVB‐N content could be attributed to many nitrogenous compounds, such as ammonia, trimethylamine, and dimethylamine produced by spoilage bacteria and endogenous digestive enzymes (Jinadasa, 2014).

A similar effect of gelatin coating enriched with cinnamon (Andevari & Rezaei, 2011) and oregano (Hosseini et al., 2016) essential oils on reducing TVB‐N content of stored rainbow trout fish. In the same context, sodium caseinate–gelatin nanofibers containing *Mentha spicata* essential oil decreased the TVB‐N content of fresh trout fillets (Eghbalian et al., 2021). Coating with gelatin/phenolics was more effective than gelatin applied alone, which can be attributed to the microbial growth inhibitory activity exerted by the phenolic compounds in the *M. oleifera* extract.

Lipid peroxidation is also an important factor limiting the sensory properties of fish products. It was reported that 1–2 mg MDA/kg is usually regarded as the limit TBARS in fresh fish fillet (Connell, 1990). Thus, TBARS were measured during the storage period (Figure 1). The initial TBARS value was 0.60 mg MDA/kg fish fillet, which was close to the value of fresh smooth‐hound shark fillets reported in an earlier study (Abdelhedi et al., 2019) (0.30 mg MDA/kg fish fillet). The TBARS values of all fish samples significantly increased (*p* ≤ .05) during the storage period. The TBARS value of the uncoated‐sample exceeded the maximum acceptable limit of 2 mg MDA/kg during 6 days of storage. Interestingly, the coated fish fillet showed significantly lower (*p* ≤ .05) TBARS content than the uncoated sample. In fact, coating with gelatin and gelatin‐MOE/W reduced the TBARS content by 54% and 70% after 6 days of storage, respectively (Figure 1). The obtained results suggest that gelatin coating contributed to prevent fish fillets oxidation, which may be improved by the addition of phenolic compounds that were effective in the lipid oxidation inhibition. Likewise, Andevari and Rezaei (2011) reported that cinnamon essential oil used in gelatin coatings reduced the lipid peroxidation of refrigerated rainbow trout fillets. The oxygen barrier properties of gelatin coatings can prevent oxidation of lipids in food products, which can be improved by antioxidant compounds (Sahraee et al., 2019). Eghbalian et al. (2021) reported similar results about TBARS content of trout fillets packaged with sodium caseinate–gelatin nanofibers containing *Me. spicata* essential oil separately or in combination with MgO. These authors suggest that the prolongation of the shelf‐life of fresh fillets was attributed to the antioxidant property of *Me. spicata* essential oil.

#### 3.2.2 Texture and color characteristics

The texture properties of the fish fillets are important sensory parameters for consumers' acceptability. Table 3 shows the values of texture parameters (strength, cohesiveness, springiness, and chewiness) in smooth‐hound shark stored during 6 days after different coating treatments. As in case of weight loss, the strength of all fish fillets significantly increased (*p* ≤ .05) during 6 days of storage, which might be due to the exudate loss (Figure 1). Interestingly, this increase was attenuated with gelatin coating. The strength of uncoated fish increased by 46%, while the strength of coated‐fish with gelatin and gelatin‐MOE/W only increased by 14% and 12%, respectively, after 6 days of storage (Table 3).

**TABLE 3:** Texture parameters of coated fish samples during storage

Parameters	Storage time (day)	Fillet	Fillet + gelatin	Fillet + gelatin‐MOE/W
Strength (g)	1	175.56 ± 0.25^bA^	189.23 ± 0.12^bB^	182.42 ± 1.71^bC^
	6	256.48 ± 1.25^aA^	215.25 ± 0.79^aB^	204.56 ± 1.82^aC^
Cohesiveness	1	0.53 ± 0.12^aA^	0.42 ± 0.03^aA^	0.45 ± 0.06^aA^
	6	0.32 ± 0.05^bA^	0.34 ± 0.06^aA^	0.39 ± 0.02^aA^
Springiness (mm)	1	2.96 ± 0.05^aA^	3.01 ± 0.45^aA^	3.11 ± 0.75^aA^
	6	1.89 ± 0.15^bB^	2.24 ± 0.62^aA^	2.86 ± 0.56^aA^
Chewiness (N × mm)	1	1.53 ± 0.36^aA^	1.45 ± 0.25^aA^	1.51 ± 0.52^aA^
	6	0.75 ± 0.11^bB^	1.11 ± 0.14^aA^	1.42 ± 0.07^aA^

*Note*: Fillet: uncoated fish fillet; fillet + gelatin: fish fillet coated with the control gelatin solution; fillet + gelatin‐MOE/W: fish fillet coated with gelatin solution enriched with *Moringa oleifera* ethanol/water extract at 20 μg/ml. Different lower case letters (^a,b^) in the same column indicate significant differences for the same sample within different days of storage (*p* ≤ .05). Different capital letters (^A,B^) indicate significant differences between samples at the same storage day (*p* ≤ .05).

Values of cohesiveness, springiness, and chewiness did not show significant differences (*p* > .05) between the coated samples or through the chilled storage within the same sample. Only slight variations throughout the chilled storage were observed within the uncoated sample. The obtained results suggest that gelatin coating did not result in important modifications in the texture properties of fish fillet during chilled storage. This seems to be supported by Gallego et al. (2020), who reported that gelatin coating enriched with antioxidant tomato by‐products did not modify the texture properties of pork meat, as compared to the uncoated sample.

The color is also a crucial factor in food quality control, since consumers rely on color to determine the freshness level of the product. Table 4 presents the color parameters of the studied fish fillet samples. The L* and b* values slightly increased during storage and were significantly higher (*p* ≤ .05) in uncoated samples than those coated with the enriched gelatin. While the a* values slightly decreased during the storage period, particularly in the uncoated sample. The chroma (C*) values, suggesting color saturation or “vivacity,” remained invariable for all samples. However, the total difference (∆E) in color showed significant differences (*p* ≤ .05) between the control and the coated samples after 6 days of storage. The ΔE* determination is used to predict the differences in perception capacity: Δ*E* < 1.5 = slightly distinct; 1.5 < Δ*E* <3.0 = distinct; and Δ*E* > 3.0 = very distinct (Adekunte et al., 2010). Similarly, Feng et al. (2016) reported that coating with fish gelatin combined with chitosan maintained the color of golden pomfret fillet during chilled storage. In fact, lower total color changes in coated samples were measured in comparison to the uncoated sample. The discoloration of fish fillet during storage or processing essentially results from the oxidation of myoglobin, as well as its reaction with other muscle components (Antoniewski & Barringer, 2010). The color changes are mainly produced for dark‐fleshed fish species, but considering that dogfish are white fish, keeping their color white may have contributed to their better appearance. Thus, the gelatin coating, and in particular the one enriched with *M. oleifera* extract, improved the color preservation of the fillet surface, probably by reducing changes in its texture and protecting it from the environment.

**TABLE 4:** Color of coated fish samples during storage

Storage time (day)						
	1			6		
	Fillet	Fillet + gelatin	Fillet + gelatin‐MOE/W	Fillet	Fillet + gelatin	Fillet + gelatin‐MOE/W
L*	61.73 ± 0.15^bA^	61.59 ± 0.59^bA^	61.02 ± 0.47^aA^	63.98 ± 0.37^aA^	62.91 ± 0.44^aA^	61.78 ± 0.60^aB^
a*	2.58 ± 0.23^aA^	2.89 ± 0.24^aA^	2.90 ± 0.14^aA^	1.02 ± 0.03^bB^	2.48 ± 0.44^aA^	2.58 ± 1.08^aA^
b*	10.77 ± 0.02^bA^	10.25 ± 0.12^aA^	10.12 ± 0.48^aA^	12.75 ± 0.24^aA^	11.01 ± 0.65^aA^	10.78 ± 0.53^aA^
c*	13.35 ± 0.23^aA^	13.14 ± 0.14^aA^	13.02 ± 0.22^aA^	13.77 ± 0.10^aA^	13.49 ± 0.72^aA^	13.36 ± 0.44^aA^
∆*E*	–	0.62 ± 0.13^bB^	1.01 ± 0.09^bA^	3.37 ± 0.13^A^	1.20 ± 0.11^aB^	0.05 ± 0.13^aC^

*Note*: Fillet: uncoated fish fillet; fillet + gelatin: fish fillet coated with the control gelatin solution; fillet + gelatin‐MOE/W: fish fillet coated with gelatin solution enriched with *Moringa oleifera* ethanol/water extract at 20 μg/ml. Different lower case letters (^a,b^) in the same row indicate significant differences for the same sample within different days of storage (*p* ≤ .05). Different capital letters (^A,B,C^) in the same row indicate significant differences between samples at the same storage day (*p* ≤ .05).

#### 3.2.3 Microbiological analysis

**FIGURE 2:**
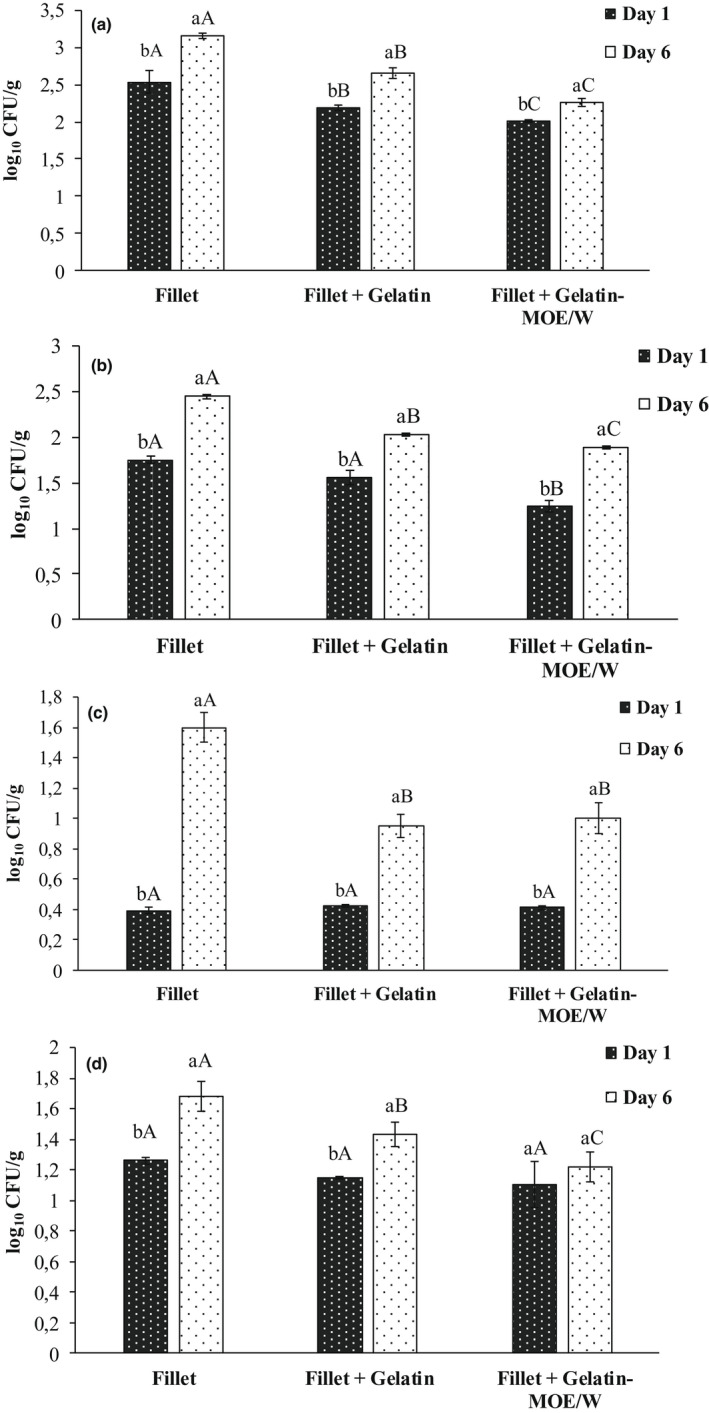
Changes in (a) mesophilic, (b) psychrophilic, (c) lactic acid, and (d) H_2_S‐producing bacteria of coated fish samples during storage. Fillet: uncoated fish fillet; fillet + gelatin: fish fillet coated with the control gelatin solution; fillet + gelatin‐MOE/W: fish fillet coated with gelatin solution enriched with *Moringa oleifera* ethanol/water extract at 20 μg/ml. Different lower case letters (^a,b,c^) indicate significant differences for the same sample within different days of storage (*p* ≤ .05). Different capital letters (^A,B,C^) indicate significant differences between samples at the same storage day (*p* ≤ .05)

Figure 2 shows the effect of coating on microbial growth of fish fillet samples. The total mesophilic bacterial count is an important indicator to estimate the shelf‐life, postprocessing contamination, and quality of the fish fillet. The psychrophilic bacteria are also responsible for fish spoilage during refrigerated storage. The initial total count of mesophilic (Figure 2a) and psychrophilic (Figure 2b) bacteria in fish fillets was between 2.01 and 2.54 log_10_ CFU/g, respectively, which was much lower than the threshold of 7.0 log_10_ CFU/g for the maximum allowable limit of fish (Swanson, 2011). An increase in mesophilic and psychrophilic bacterial count was measured from day 1 to day 6 for all fish samples. Coating with gelatin significantly reduced (*p* ≤ .05) the number of these bacteria during storage. Moreover, coating with gelatin‐MOE/W resulted in a reduction of ~1 log10 CFU/g in the number of mesophilic bacteria. Likewise, the initial count of LAB in uncoated fish fillets was 1.26 log_10_ CFU/g (day 1) and reached 1.68 log_10_ CFU/g at day 6 of refrigerated storage (Figure 2c). The LAB growth in the uncoated sample was significantly higher (*p* ≤ .05) than the coated samples over the storage period. Similarly, the count of H_2_S‐producing bacteria (Figure 2d) of fillets coated with gelatin or gelatin‐MOE/W remained significantly lower (*p* ≤ .05) than that of uncoated sample during the storage period. Interestingly, the gelatin‐MOE/W coating showed a stronger inhibitory effect on the all analyzed microbial populations, compared to uncoated or gelatin‐coated samples.

Similar results were reported by Eghbalian et al. (2021) who developed sodium caseinate–gelatin nanofibers containing *Me. spicata* essential oil. These authors indicated that at the end of the storage period (day 13), trout samples packaged in such active material had microbial populations (Enterobacteriaceae and mesophilic, psychrotrophic, lactic acid, and H_2_S‐producing bacteria) lower than those of the control group. Shahbazi et al. (2021) also reported that carboxymethyl cellulose–gelatin nanofibrous films encapsulated with *Mentha longifolia* essential oil successfully extended the shelf‐life of peeled freshwater prawns to 14 days of refrigerated storage. Furthermore, Hosseini et al. (2016) reported an enhanced reduction of psychrophilic bacterial count of rainbow trout fillet after coating with fish gelatin containing oregano essential oil. The protective effect of the fish gelatin coating, forming a protein biofilm surrounding the fish fillet, can be attributed to its role as a barrier against oxygen diffusion and subsequently bacterial proliferation. Besides, the antimicrobial activity of MOE/W extract added in the film could enhance the antimicrobial property of edible gelatin coating, which could be an effective way to extend the storage period of fish fillet.

#### 3.2.4 Sensory analysis

**FIGURE 3:**
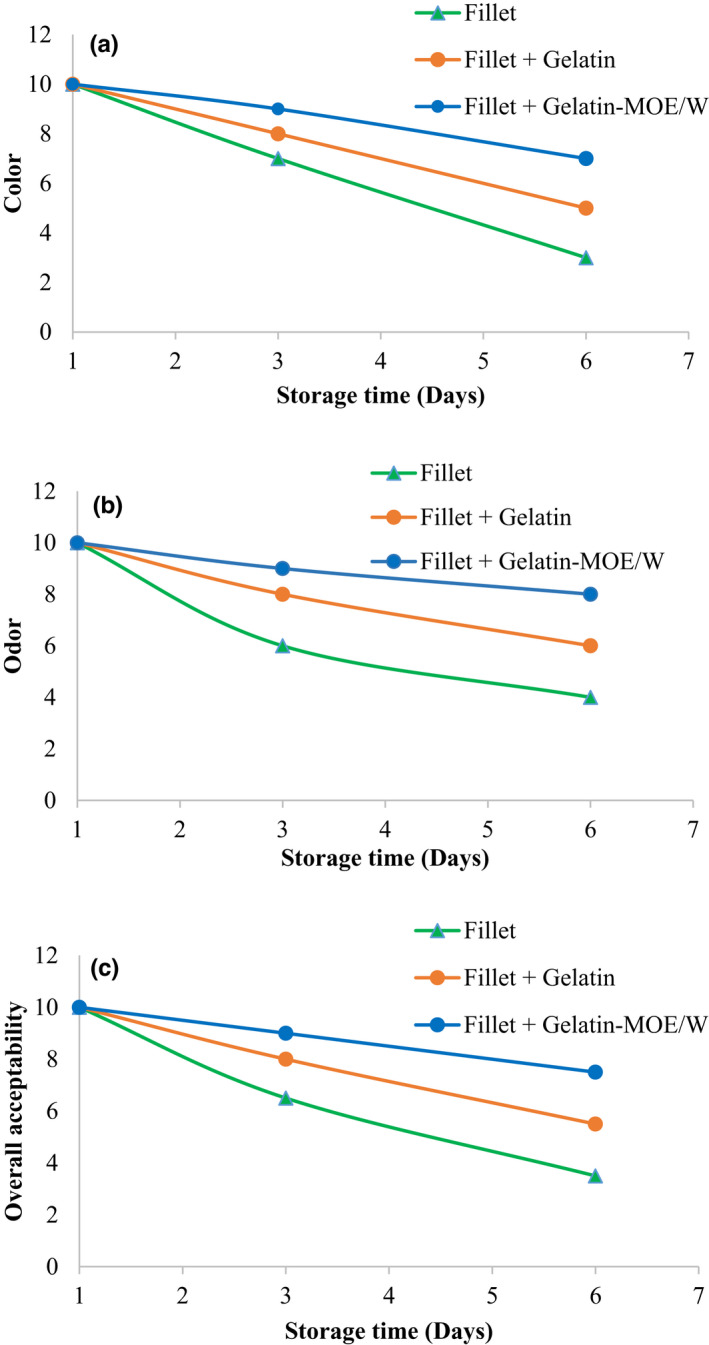
Changes in sensory attributes (a, odor; b, color; c, overall acceptability) of fresh coated fish samples during storage. Fillet: uncoated fish fillet; fillet + gelatin: fish fillet coated with the control gelatin solution; fillet + gelatin‐MOE/W: fish fillet coated with gelatin solution enriched with *Moringa oleifera* ethanol/water extract at 20 μg/ml

The sensory results (odor, color, and general acceptability) of fresh fillets are shown in Figure 3a–c. Sensory properties of all samples decreased throughout the storage period for all samples. As compared to the control group, coated samples showed higher scores for all attributes. The gelatin‐MOE/W coated fish fillet had the highest sensory scores in odor, color, and overall acceptability over the storage period. In this regard, it was reported that trout fillets coated with sodium caseinate nanofibers added with *Me. spicata* essential oil showed better sensory properties compared to the control sample (Eghbalian et al., 2021).

## 4 CONCLUSION

Edible coatings enriched with natural bioactive compounds are increasingly in demand in the food industry. This study showed that fish fillet coated with a combination of fish gelatin and *M. oleifera* extract contributed to delay its deterioration during chilled storage. Furthermore, the gelatin coating enriched with *M. oleifera* extract improved the color preservation of the fillet surface and reduced changes in its texture properties. The obtained results may be due to the functionality of phenolic compounds in *M. oleifera* extract, which exerted interesting antioxidant and antibacterial potential, in addition to the barrier properties of gelatin. Therefore, gray triggerfish gelatin fortified with *M. oleifera* leaf extract plays a beneficial role, as an edible coating agent, in extending the shelf life of the fish products.

## CONFLICT OF INTEREST

The authors declare that they have no conflict of interest.

## Data Availability

All authors confirm that the data supporting the findings of this study are available within the article.
